# Structured Manual Muscle Testing of the Lower Limbs

**DOI:** 10.21315/mjms2023.30.5.17

**Published:** 2023-10-30

**Authors:** Xiao Yi Lim, Jonathan Kee Chi Wong, Zamzuri Idris, Abdul Rahman Izaini Ghani, Sanihah Abdul Halim, Jafri Malin Abdullah

**Affiliations:** 1 Department of Neurosciences, School of Medical Sciences, Universiti Sains Malaysia, Kelantan, Malaysia; 2 Hospital Universiti Sains Malaysia, Universiti Sains Malaysia, Kelantan, Malaysia; 3 Department of Neurosurgery, Hospital Raja Permaisuri Bainun, Perak, Malaysia; 4 Department of Neurosurgery, Hospital Umum Sarawak, Sarawak, Malaysia; 5 Neurology Unit, Department of Internal Medicine, School of Medical Sciences, Universiti Sains Malaysia, Kelantan, Malaysia; 6 Brain and Behaviour Cluster, School of Medical Sciences, Universiti Sains Malaysia, Kelantan, Malaysia

**Keywords:** manual muscle testing, strength of lower limb, neurological examination

## Abstract

An accurate and reliable neurological examination is pivotal in diagnosing patients with neurological and neurosurgical conditions. Despite the advancement of neuroscientific knowledge and the ever-progressing technologies and modalities that are being adopted to help achieve the challenge of accurate diagnosis, the neurologic examination is still crucial in both ambulatory and emergency settings. It provides the physician a tool to recognise neurologic involvement in certain disease states, and thereby allow proper work-up and treatment for patients. A basic neurologic examination can be performed rapidly with practice. Manual muscle testing of the lower limbs was carried out in accordance with a bedside clinical examination involving a clinical personnel examiner and a patient. This testing was performed in a rostro-caudal manner, starting from the hip and progressing to the toes. The neurological exam can be intimidating to perform for a lot of physicians. Deficiencies in accurate muscle testing have always presented a challenge for medical students and clinicians. By referring to the examination methods mentioned in our text and with the help of related video, it is our aim to improve the quality of neurological examination among medical personnel so that diseases may be recognised and managed earlier in their course.

## Introduction

Neurological examination is used as an investigative tool for a patient when a neurological abnormality is found on screening, or when an abnormality can be expected from the patient’s history. The aim of examination is to determine whether there is an abnormality, what the nature and extent of the abnormality is, and whether there are any associated abnormalities. The methods of neurological examination have evolved gradually. In the late 1800s, Wilhelm Erb, Joseph Babinski, William Gowers and others developed the neurologic exam as we know it today. They described examination techniques in their articles and texts about neurology. As knowledge of the nervous system and its diseases grew, the complexity and length of the neurologic exam increased. The examination spans from assessment of higher mental function to examination of the motor and sensory system. Examination of the motor system allows the degree of motor function impairment to be quantified and often allows differentiation between central and peripheral lesions. The fundamental elements of the examination include muscle appearance, muscle strength (power), tone and reflexes.

The importance of using correction technique when testing muscle strength cannot be overemphasised. When testing individual muscle strength, one should be sure to position the limb in such a way as to permit the muscle being examined to act directly and to minimise the recruitment of other muscles with similar a function. Methods of grading muscle power in different muscle groups can vary and so a generally accepted technique has to be adopted to minimise discrepancies between examiners.

Through referencing to the examination methods that were mentioned in our text and with the help of related video, it is our aim to improve the quality of neurological examination among medical personnel in order to recognise and manage diseases earlier in their course. The objective of this article is thus to delineate the correct methods for testing the muscles of the hip, knee, ankle and foot.

## Methods

### Setting

The manual muscle testing of the lower limbs was performed in the Student Resource Centre of Hospital Universiti Sains Malaysia (HUSM), Kubang Kerian. HUSM is a tertiary teaching university hospital located in the state of Kelantan, Malaysia.

### Equipment and Personnel

The examination was conducted in a such way as to mimic a bedside clinical examination with the participation of an examining doctor and a patient. The examination was performed with the patient sitting or lying on the examination bed. An adjustable bed was used during the examination. The entire examination process was recorded with a video recorder.

### Video

The video recorded during the manual muscle testing was edited to incorporate slides explaining each of the methods of examination followed by the video of the examination itself. The link to the video is available at https://youtu.be/xnGcE9CchYo

The manual muscle testing is performed in accordance with the Medical Research Council (MRC) grading for muscle power which grades muscle power as follow:

0 - No contraction
1 - Flicker or trace of contraction
2 - Active movement, with gravity eliminated
3 - Active movement against gravity
4 - Active movement against gravity and resistance
5 - Normal power

### Hip

- Hip flexion
- Hip flexion, abduction and external rotation with knee flexion
- Hip extension
- Hip extension test to isolate gluteus maximus
- Hip extension tests modified for hip flexion tightness
- Supine hip extension test
- Hip abduction
- Hip abduction from flexed position
- Hip adduction
- Hip external rotation
- Hip internal rotation

#### i) Hip flexion

The primary muscles tested are psoas major and iliacus.

##### Grading

For examination for Grades 5, 4 and 3, patient sits with their thighs fully supported on the table and their legs hanging over the edge of the table. Patient may use arms to provide trunk stability by grasping the table edge or putting their hands on each side of the table.

Examiner stands next to the limb to be tested. The patient is asked to lift their thigh off the table. If adequate range is present (thigh clears the table), maximum resistance is applied at midrange (Grade 5) over the distal thigh just proximal to the knee joint, being careful not to grasp the thigh.

Patient flexes hip to end of range, clearing the table and maintaining neutral rotation. Patient then brings their hip to midrange and holds that position against resistance, which is given in a downward direction towards the floor.

Grade 5: Holds test position against maximal resistance.

Grade 4: Holds test position against strong to moderate resistance. There may be some ‘give’ with maximum resistance.

Grade 3: Completes test range and holds the position without resistance.

For examination for Grade 2, patient lies in side-lying position with limb to be tested uppermost and supported by examiner. Trunk in neutral alignment; lowermost limb may be flexed for stability.

Examiner stands behind patient. Test limb is cradled in one arm with hand support under the slightly flexed knee. The examiner’s opposite hand maintains patient’s trunk alignment at hip.

Patient flexes hip with supported limb. Knee is permitted to flex to prevent hamstring tension.

Grade 2: Completes range of motion in side-lying position.

For examination for Grades 1 and 0, patient lies supine.

Examiner stands at side of the limb to be tested. The test limb is supported under calf with one hand behind knee. The examiner’s free hand palpates the muscle just distal to the inguinal ligament on the medial side of the sartorius.

Patient attempts to flex hip.

Grade 1: Palpable contraction but no visible movement.

Grade 0: No discernible palpable contraction of muscle.

#### ii) Hip flexion, abduction and external rotation with knee flexion

The primary muscle tested is sartorius.

##### Grading

For examination for Grades 5, 4 and 3, patient is in short sitting position with thighs supported on the table and legs hanging over the side. Arms may be used for support.

Examiner stands lateral to tested leg. Patient is asked to flex, abduct and externally rotate the hip, with the knee flexed. If adequate range is present, place one hand on the lateral side of knee, the other hand over the medial-anterior surface of distal leg. Proximal hand resists hip flexion and abduction (in a downward and inward direction) and hand in distal leg resists hip external rotation and knee flexion (up and outward).

Patient flexes, abducts and externally rotates the hip while maintaining knee flexion.

Grade 5: Holds test position against maximal resistance; limb does not ‘give’.

Grade 4: Tolerates moderate to strong resistance while maintaining position.

Grade 3: Completes movement and holds test position without resistance.

For examination for Grade 2, patient is in supine position. Heel of limb to be tested is placed on contralateral shin.

Examiner stands at side of limb to be tested. Limb is supported as necessary to maintain alignment.

Patient slides test heel upward along shin towards knee.

Grade 2: Completes desired movement.

For examination for Grades 1 and 0, patient is in supine position.

Examiner stands on side to be tested. Test limb is cradled under calf with examiner’s hand supporting the limb behind the knee. Examiner’s opposite hand palpates sartorius on medial side of thigh where muscle crosses the femur. Examiner may palpate near the muscle origin just below the anterior superior iliac spine (ASIS).

Patient slides heel up shin towards knee.

Grade 1: Slight contraction of muscle, with no visible movement.

Grade 0: No discernible palpable contraction.

#### iii) Hip extension

The primary muscles tested are gluteus maximus and hamstrings.

##### Grading

For examination for Grades 5, 4 and 3, patient is positioned prone. Arms may be at the side of the body or abducted to hold the sides of the table.

Examiner stands at level of pelvis on side of limb to be tested. The patient is asked to lift the leg off the table as high as possible, while keeping the knee straight. If sufficient range is achieved, hand providing resistance is placed on posterior leg just above the ankle. The opposite hand may be used to stabilise or maintain pelvis alignment in the area of posterior superior spine of ilium.

Patient extends hip through entire available range of motion. Resistance is given downwards towards the floor.

Grade 5: Holds test position against maximal resistance.

Grade 4: Holds test position against strong to moderate resistance.

Grade 3: Completes range and holds the position without resistance.

For examination for Grade 2, patient is in side-lying with test limb uppermost. The knee is positioned straight and is supported by examiner. Lowermost limb is flexed for stability.

Examiner stands behind patient at thigh level. Examiner supports test limb just below the knee, cradling the leg. Opposite hand is placed over the pelvic crest to maintain pelvic and hip alignment.

Patient extends hip through full range of motion.

Grade 2: Completes available range of motion in side-lying position.

For examination for Grades 1 and 0, patient in prone position.

Examiner stands at level of pelvis on side to be tested. Hamstrings (with fingers deep into the tissue) are palpated at the ischial tuberosity. Gluteus maximus is palpated with deep finger pressure over the centre of the buttocks including the upper and lower fibres.

Patient attempts to extend hip in prone position or tries to squeeze buttocks together.

Grade 1: Palpable contraction of gluteus maximus but no visible joint movement. Contraction of gluteus maximus will result in narrowing of the gluteal crease.

Grade 0: No discernible palpable contraction.

#### iv) Hip extension test to isolate gluteus maximus

For examination for Grades 5, 4 and 3, patient is positioned prone with knee flexed to 90°, hip abducted and externally rotated.

Examiner stands at the level of pelvis on the side to be tested. Patient is asked to lift the thigh off the plinth as high as possible, while bending the knee. If sufficient range is achieved, resistance is placed over posterior thigh just above the knee. The opposite hand may stabilise or maintain the pelvic alignment.

Patient extends abducted and externally rotated hip through available extension range, maintaining knee flexion. Resistance is given in a straight downward direction (towards floor).

Grade 5: Holds test position against maximal resistance.

Grade 4: Holds test position against strong to moderate resistance.

Grade 3: Completes available range of motion and holds test position but without resistance.

For examination for Grade 2, patient in side-lying position with test limb uppermost. Knee is flexed and supported by examiner. Lowermost hip and knee should be flexed for stability. Examiner provides stabilisation and alignment through the uppermost hip by assuring pelvis and hip are in line with shoulder.

Examiner stands at level of pelvis behind the patient. Examiner cradles uppermost leg with forearm. Other hand stabilises pelvis in neutral alignment at the iliac crest.

Patient extends hip with supported knee flexed.

Grade 2: Completes available range of motion in side-lying position.

For examination for Grades 1 and 0, patient is in prone position.

Examiner stands at level of pelvis on side to be tested. Hamstrings (with fingers deep into the tissue) are palpated at the ischial tuberosity. Gluteus maximus is palpated with deep finger pressure over the centre of the buttocks including the upper and lower fibres.

Patient attempts to extend hip in prone position or tries to squeeze buttocks together.

Grade 1: Palpable contraction of gluteus maximus but no visible joint movement. Contraction of gluteus maximus will result in narrowing of the gluteal crease.

Grade 0: No discernible palpable contraction.

#### v) Hip extension tests modified for hip flexion tightness

For examination for Grades 5, 4 and 3, patient leans over the table with hips flexed so that the ASIS is ‘hooked’ on the end of table. Arms are used to ‘hug’ the table for support. Knee of non-test limb should be flexed to allow test limb to rest on floor at start of test.

Examiner stands at the side of limb to be tested. Patient is asked to lift his leg towards the ceiling. If sufficient range is achieved, hand is placed over the posterior thigh just above the knee for resistance. The opposite hand stabilises the pelvis laterally to maintain hip and pelvis posture.

Patient extends hip through available range, but hip extension range is less when knee is flexed. Resistance is applied downward towards the floor.

Grade 5: Holds test position against maximal resistance.

Grade 4: Holds test position against strong to moderate resistance.

Grade 3: Completes available range and holds test position without resistance.

For examination for Grades 2, 1 and 0, patient positioned side-lying on the table. Knee straight and supported by examiner. Lowermost limb is flexed for stability.

Examiner stands behind patient at thigh level. Examiner supports test limb just below the knee, cradling the leg. Opposite hand is placed over the pelvic crest to maintain pelvic and hip alignment.

Patient extends hip through full range of motion.

Grade 2: Completes available range of motion in side-lying position.

For examination for Grades 1 and 0, patient in prone position.

Examiner stands at level of pelvis on side to be tested. Hamstrings (with fingers deep into the tissue) are palpated at the ischial tuberosity. Gluteus maximus is palpated with deep finger pressure over the centre of the buttocks including the upper and lower fibres.

Patient attempts to extend hip in prone position or tries to squeeze buttocks together.

Grade 1: Palpable contraction of gluteus maximus but no visible joint movement. Contraction of gluteus maximus will result in narrowing of the gluteal crease.

Grade 0: No discernible palpable contraction.

#### vi) Supine hip extension test

##### Grading

For examination for Grades 5, 4, 3 and 2, patient is in supine position with heels off end of table and arms folded across chest or abdomen.

Examiner stands at the end of table and lifts patient’s leg to at least 65° of flexion. Patient’s hip range is determined by measuring from heel to table (35 in from heel to table is approximately 65° of flexion). Examiner is in a squat position with knees and hips bent and elbow straight). Both hands are cupped under the heel and patient is asked to push into examiner’s hands, keeping the knee straight and hip locked. Try to raise the limb to the initial height measured.

Patient presses heel into examiner’s cupped hands, attempting to maintain full extension as the examiner raises the limb approximately 35 in from the table.

Grade 5: Hip locks in neutral (full extension) throughout this test. Pelvis and back elevate as one and are locked as the examiner raises the limb. The opposite limb will rise involuntarily, illustrating a locked pelvis.

Grade 4: Hip flexes before pelvis and back elevate and lock as the limb is raised by the examiner. Hip flexion should not exceed 30º before locking occurs. The other leg will rise involuntarily, but will have some hip flexion because the pelvis is not fully locked.

Grade 3: Full flexion of hip to the end of the straight-leg raising range (65º hip flexion) with little or no elevation of the pelvis, demonstrated by the other leg remaining on table. Examiner feels strong resistance throughout the test.

Grade 2: Hip flexes fully with only minimal resistance felt (ensure the resistance felt exceeds the weight of the limb).

There is no Grade 1.

Grade 0: Hip flexes fully with no active resistance felt by the examiner as limb is raised. Examiner perceives that resistance is due to leg weight only.

#### vii) Hip abduction

The primary muscles tested are gluteus medius and gluteus minimus.

For examination for Grades 5, 4 and 3, patient is positioned side-lying with test leg uppermost. Test is started with hip slightly extended beyond the midline and pelvis rotated slightly forward. Lowermost leg is flexed for stability.

Examiner stands behind patient and asks patient to lift the leg as high as possible, giving verbal and tactile clues to keep the pelvis from rotating the hip. If sufficient range is achieved, apply resistance at the ankle. If the patient is unable to hold limb against resistance at the ankle, resistance is then applied at the lateral knee.

Patient abducts hip through the available range of motion without flexing the hip or rotating it in either direction. Resistance is given in a straight downward direction.

Grade 5: Holds test position against maximal resistance at the ankle.

Grade 4: Holds test position against strong to moderate resistance at the ankle (limb cannot hold the position) or with maximum resistance given at the knee.

Grade 3: Completes range of motion and holds test position without resistance. Hip should not flex into frontal plane or rotate.

For examination for Grade 2, patient is placed in supine position.

Examiner stands on side of tested limb with one hand supporting and lifting the limb by holding it under the ankle to raise limb just enough to decrease friction. This hand offers no resistance nor assistance to the movement. The other hand palpates the gluteus medius just proximal to the greater trochanter of the femur.

Patient abducts hip through available range.

Grade 2: Completes range of motion supine with no resistance and minimal to zero friction.

For examination for Grades 1 and 0, patient is in supine position.

Examiner stands at the side of tested limb at level of thigh. One hand supports the limb under the ankle just above the malleoli, without resistance nor assistance. Palpate the gluteus medius on the lateral aspect of hip just above the greater trochanter. The weight of the opposite limb stabilises the pelvis. It is not necessary therefore to use a hand to manually stabilise the contralateral limb.

Patient attempts to abduct hip.

Grade 1: Palpable contraction of gluteus medius but no movement of the part.

Grade 0: No discernible contractile activity.

#### viii) Hip abduction from flexed position

The primary muscle tested is tensor fascia latae.

##### Grading

For examination for Grades 5, 4 and 3, patient is in side-lying position, lying across the lowermost limb with their foot resting on the table and their uppermost limb (test limb) flexed to 45°.

Examiner stands behind patient at level of pelvis. Patient is asked to flex hip and lift leg to 30°. If successful, resistance is placed on lateral surface of thigh just above the knee. Hand providing stabilisation is placed on the crest of the ilium.

Patient abducts hip through approximately 30° of motion. Resistance is given downward towards floor from the lateral surface of distal femur.

Grade 5: Holds test position against maximum resistance.

Grade 4: Holds test position against strong to moderate resistance.

Grade 3: Completes movement; holds test position but without resistance.

For examination for Grade 2, patient is in long-sitting position, supporting trunk with hands placed behind body on table. Trunk may lean backward up to 45° from vertical.

Examiner stands at side of tested limb. One hand supports the limb under the ankle to reduce friction with the surface as patient moves but the hand should neither resist nor assist movement. The other hand palpates the tensor fascia latae on the proximal anterolateral thigh where it inserts into the iliotibial band.

Patient abducts hip through 30° range.

Grade 2: Completes hip abduction to 30°.

For examination for Grades 1 and 0, patient is in long-sitting position.

Examiner uses one hand to palpate the insertion of tensor at the lateral aspect of knee. The other hand palpates the tensor on the anterolateral thigh.

Patient attempts to abduct hip.

Grade 1: Palpable contraction of tensor fibres but no limb movement.

Grade 0: No discernible palpable contractile activity.

#### ix) Hip adduction

The primary muscles tested are adductors magnus, brevis and longus, pectineus and gracilis.

##### Grading

For examination for Grades 5, 4 and 3, patient is in side-lying position with test limb (lowermost) resting on table.

Examiner stands behind patient at knee level and support the uppermost limb (non-test limb) in 25° of abduction with forearm and the hand supporting the limb on the medial surface of knee. Alternatively, the upper limb can be placed on a padded stool straddling the test limb and approximately 9 in–12 in high. Patient is asked to lift the bottom leg to the uppermost one. If successful, hand is placed on the medial surface of the distal femur of the lower limb, just proximal to knee joint to give resistance. Direct resistance straight downward towards the table.

Patient adducts hip until lower limb contacts upper limb.

Grade 5: Holds test position against maximal resistance.

Grade 4: Holds test position against strong to moderate resistance.

Grade 3: Completes full range; holds test position without resistance.

For examination for Grade 2, patient is in supine position with non-test limb positioned in some abduction to prevent interference with motion of test limb.

Examiner stands at side of test limb at knee level. One hand supports the ankle and elevates it slightly from the table surface to decrease friction as the limb moves across the table. The opposite hand palpates the adductor mass on the inner aspect of the proximal thigh.

Patient adducts hip without rotation. Toes stay pointed towards the ceiling.

Grade 2: Adducts limb through full range with gravity minimised.

Grade 1: Palpable contraction, no limb movement.

Grade 0: No discernible palpable contraction.

#### x) Hip external rotation

The primary muscles tested are obturators internus and externus, gemelli superior and inferior, piriformis, quadratus femoris, and gluteus maximus (posterior).

##### Grading

For examination for Grades 5, 4, 3 and 2, patient is in short-sitting position with thighs fully supported on table and legs hanging over the edge of the table.

Examiner sits on a low stool or kneel beside limb to be tested. Patient is asked to turn the leg in. If sufficient range is present, leg is positioned in mid position between internal and external rotation. Hand is placed on the medial aspect of ankle just above the malleolus to provide resistance. The other hand is contoured over the lateral aspect of the distal thigh just above the knee to provide counter-pressure. The two forces are applied in counter-directions for this rotary motion.

Patient externally rotates the hip.

Grade 5: Holds test position in midrange against maximal resistance.

Grade 4: Holds test position in midrange against strong to moderate resistance.

Grade 3: Able to complete full range of motion with mild to no resistance (this is a gravity-eliminated position, so if the patient is able to exert mild resistance, the effort should be graded 3)

Grade 2: Completes full range of motion without resistance. It should be ensured that gravity is not the predominant force.

For Grade 2 alternate test (if patient cannot sit), patient is placed supine with test limb in internal rotation.

Examiner stands at the side of tested limb. Examiner may need to support the limb in internal rotation because gravity tends to pull the limb into external rotation.

Patient externally rotates hip in available range of motion. One hand may be used to maintain pelvic alignment at lateral hip.

Grade 2: Completes external rotation range of motion. As hip rolls past midline, minimal resistance can be offered to offset the assistance of gravity.

For examination for Grades 1 and 0, patient in supine position with test limb placed in internal rotation and patient attempts to externally rotate the hip.

Grade 1: External rotator muscles, except for gluteus maximus, are not palpable. Presence of discernible movement.

Grade 0: No discernible palpable contraction.

#### xi) Hip internal rotation

The primary muscles tested are glutei minimus and medius and tensor fasciae latae.

##### Grading

For examination for Grades 5, 4, 3 and 2, patient is in short-sitting position with thighs fully supported on table and legs hanging over the edge of the table.

Examiner sits or kneels in front of patient and asks the patient to move their leg out, away from the other leg while maintaining hip stabilisation. If sufficient range is present, leg is positioned in mid-position between internal and external rotation. Hand is placed on lateral surface of ankle just above the malleolus to provide resistance. The other hand is contoured over the medial surface of the distal thigh just above the knee to provide counter-pressure. Stabilisation is provided in a medially directed force at the knee that counteracts the lateral resistance provided at the ankle.

Grade 5: Holds test position against maximal resistance.

Grade 4: Holds test position against strong to moderate resistance.

Grade 3: Able to complete full range of motion with mild to no resistance.

Grade 2: Able to complete full range of motion but cannot tolerate resistance. Ensure gravity is not the predominant force.

For Grade 2, alternate test in patients who cannot sit, with patient in supine with test limb in partial external rotation.

Examiner stands next to test leg and palpates the gluteus medius proximal to the greater trochanter and the tensor fasciae latae over the anterolateral hip below the ASIS.

Patient internally rotates hip through available range.

Grade 2: Holds test position. As the hip rolls inward past the midline, minimal resistance can be offered to offset the assistance of gravity.

For examination for Grades 1 and 0, patient is placed in supine position with test limb placed in external rotation. Examiner stands next to test leg.

Patient attempts to internally rotate the hip. One hand is used to palpate the gluteus medius (over posterolateral surface of hip above greater trochanter). The other hand palpates the tensor fasciae latae (on anterolateral surface of hip below ASIS).

Grade 1: Palpable contractile activity in either or both muscles.

Grade 0: No discernible contractile activity.

##### Knee

The movements of knee include:

- Knee flexion
- Knee extension

#### i) Knee flexion

##### Grading

There are three basic muscle tests for hamstrings at Grades 5 and 4. First, the aggregate of three hamstring muscles is tested for with the foot in midline. If there is any deviation (or asymmetry) in the movement, the medial and lateral hamstrings will be tested separately. The hamstrings are two joint muscles and should be tested in mid-range.

- Hamstring muscles in aggregate
- Medial hamstring test
- Lateral hamstring test

###### i) Hamstring muscles in aggregate

Patient is positioned prone with legs straight and toes hanging over the edge of the table. A towel roll is placed just above the knee for comfort.

Examiner stands next to the limb to be tested and asks the patient to flex the knee as far as possible. Observe possible tightness in the rectus femoris that may be indicated by limited knee flexion or the hip flexing. If sufficient range is present, place limb in about 45º of knee flexion (mid-range). Hand provides resistance on posterior surface of the leg just above the ankle. The other hand provides stabilisation for the hamstring tendons on the posterior thigh (optional). Firm pressure with the stabilising hand may offset any cramping of the hamstring muscles. Resistance is applied in the direction of knee extension for Grades 5 and 4.

Patient holds knee in 45° of knee flexion while maintaining leg in neutral rotation.

###### ii) Medial hamstring test

The primary muscles tested are semitendinosus and semimembranosus.

Patient lies prone with knee flexed to 45°. Leg in internal rotation (toes pointing toward midline). Examiner resists knee flexion at ankle using a downward and outward force.

Patient flexes knee, maintaining leg in internal rotation (heel toward therapist, toes pointing toward midline).

###### iii) Lateral hamstring test

The primary muscle tested is biceps femoris.

Patient lies prone with knee flexed to 45°. Leg in external rotation (toes pointing laterally).

Examiner resists knee flexion at ankle using a downward and inward force.

Patient flexes knee, maintaining leg in external rotation (heel away from therapist, toes pointing towards therapist).

Grade 5 for all three tests: Holds test position against maximal resistance.

Grade 4 for all three tests: Holds test position against strong to moderate resistance.

Grade 3 for all three tests: Completes full range of motion without external resistance.

For examination for Grade 2, patient is positioned side-lying with test limb (uppermost limb) supported by examiner or resting on suitable height stool. Lower limb is flexed for stability.

Examiner stands behind patient at knee level. One arm is used to cradle thigh, providing hand support at medial side of knee, while the other hand supports the leg at the ankle, just above the malleolus.

Patient flexes knee through available range of motion.

Grade 2: Completes available range of motion in side-lying position, with gravity eliminated.

To examine Grades 1 and 0, patient is positioned prone. Limbs are straight with toes extending over end of table. Knee is partially flexed and supported at ankle by therapist.

Examiner stands next to the test limb at knee level with one hand supporting the flexed limb at the ankle while the other hand palpates both the medial and lateral hamstring tendons just above the posterior knee.

Patient attempts to flex knee.

Grade 1: Tendons become prominent, but no visible movement occurs.

Grade 0: No discernible contraction of the muscles; tendons do not stand out.

#### ii) Knee extension

The primary muscle tested is quadriceps femoris.

##### Grading

For examination for Grades 5, 4 and 3, patient is positioned in short-sitting position. A towel roll is placed under the patient’s distal thigh for comfort. Patient may rest hands on the table on either side of the body for stability or may grasp the table edge. Patient is allowed to lean slightly backward to relieve hamstring muscle tension but is not allowed to hyperextend the knee because this may lock the knee into position, thus masking weakness.

Examiner stands at the side of limb to be tested and asks the patient to straighten the knee. If sufficient range is present, the knee is positioned approximately 15° of knee flexion. The palm of hand provides resistance over the anterior surface of the distal leg just above the ankle, using a straight arm technique because of the potential strength of these muscles. For Grades 5 and 4, resistance is applied in a downward direction towards the floor.

Patient extends knee through available range of motion but not beyond 0°.

Grade 5: Holds test position against maximal resistance. Examiner should not be able to break the Grade 5 knee extensors.

Grade 4: Holds test position against strong to moderate resistance.

Grade 3: Completes available range, including the last 15°.

For examination for Grade 2, patient is positioned side-lying with test limb uppermost. Lowermost limb may be flexed for stability. Limb to be tested is held in about 90º of knee flexion. The hip should be in full extension.

Examiner stands behind patient at knee level. One arm of the examiner cradles the test limb around the thigh with the hand supporting the underside of the knee. The other hand holds the leg just above the malleolus.

Patient extends knee through the available range of motion. Examiner does not assist or resist the patient’s voluntary movement.

Grade 2: Completes available range of motion.

For examination for Grades 1 and 0, the patient is positioned supine.

Examiner stands next to the limb to be tested at knee level with one hand placed on the quadriceps tendon just above the knee with the tendon ‘held’ gently between the thumb and fingers for palpation. Examiner may also palpate the patellar tendon just below the knee.

Patient attempts to extend the knee.

Grade 1: Contractile activity can be palpated in muscle through the tendon. No joint movement occurs.

Grade 0: No discernible contractile activity.

##### Ankle

- Ankle plantar flexion
- Foot dorsiflexion and inversion
- Foot inversion
- Foot eversion with plantar flexion

#### i) Ankle plantar flexion

The primary muscles tested are gastrocnemius and soleus.

##### Grading

For examination for Grades 5, 4 and 3, patient is positioned standing on the limb to be tested with knee extended, facing a wall. Fingers can be placed on the wall above shoulder height for external support. Alternatively, no more than one or two fingers should be used on a table (or other horizontal surface).

Examiner assesses range of motion of the ankle to assure sufficient range is present. Demonstrate heel rise to patient. Next, stand or sit with a lateral view of the test limb to ascertain height of heel rise. Ask patient to lift heel while keeping knee straight. If patient can clear the floor by 2 inches, ask the patient to continue lifting the heel until the patient can no longer achieve 1 inch of rise. This is when the test is terminated. Patient should not bear weight through arms.

Patient raises heel from floor consecutively through maximum available range at a rate of one rise every 2 seconds until patient no longer achieves 50% of initial plantar range.

Grade 5: Successfully completes 25 heel rises through full range of motion without a rest between rises.

Grade 4: Completes between 2 and 24 heel rises of at least 50% of initial heel raise height at a consistent rate of one rise every 2 seconds using correct form in all repetitions.

Grade 3: Able to hold body weight once in a heel-up position, but unable to raise body weight from neutral more than one time.

For examination for Grade 2, patient is positioned prone with feet off end of table.

Examiner stands at the foot of patient and asks patient to flex and extend ankle to assure sufficient range is present. Hand giving resistance is placed against the plantar surface at the level of metatarsal heads with foot in 80° of dorsiflexion.

Patient plantar flexes ankle against manual resistance.

Grade 2: Holds test position against maximal manual resistance.

For examination for Grades 1 and 0, patient is positioned prone with feet off end of table.

Examiner stands at end of table in front of foot to be tested. One hand palpates gastrocnemius-soleus activity by monitoring tension in the Achilles’ tendon just above the calcaneus. The muscle bellies of the two muscles may also be palpated.

Patient attempts to plantar flex the ankle.

Grade 1: Able to move through partial range. Contractile activity may be palpated in muscle bellies. The best location to palpate the gastrocnemius is at midcalf with thumb and fingers on either side of the midline but above the soleus. Palpation of soleus is best done on the posterolateral surface of the distal calf.

Grade 0: No discernible palpable contraction.

#### ii) Foot dorsiflexion and inversion

The primary muscle tested is tibialis anterior.

##### Grading

Patient is positioned supine.

Examiner stands at the foot of patient with patient’s heel resting on table. Patient brings the foot up and in, towards the body. If sufficient range exists, place hand providing resistance on the medial aspect of the foot over the first ray. Resistance if provided down and out.

Patient dorsiflexes ankle and inverts foot, keeping toes relaxed.

Grade 5: Holds test position against maximal resistance.

Grade 4: Holds test position against strong to moderate resistance.

Grade 3: Completes available range of motion without resistance.

Grade 2: Completes only a partial range of motion.

Palpate the tendon of tibialis anterior on the anteromedial aspect of ankle at about the level of the malleoli. Alternatively, palpate the muscle for contractile activity over its belly just lateral to the ‘shin’.

Grade 1: Contractile activity is detected in the muscle or the tendon will ‘stand out’. No joint movement.

Grade 0: No discernible palpable contraction.

#### iii) Foot inversion

The primary muscle tested is tibialis posterior.

##### Grading

For examination for Grades 5, 4, 3 and 2, patient is in supine positionwith ankle in slight plantar flexion.

Examiner sits on a low stool in front of patient or on side of test limb (anti-gravity position). With patient’s heel resting on examiner’s thigh, ask the patient to move the foot down and in. Movement is performed passively if required. If sufficient active range exists, place stabilising hand on the posterior calf just above the malleoli. The majority of resistance is towards forefoot abduction (up and out direction). Hand providing resistance is placed on the foot with the hand providing resistance over the medial side of the forefoot.

Patient inverts foot through available range of motion.

Grade 5: Holds the test position against maximal resistance.

Grade 4: Holds the test position against strong to moderate resistance.

Grade 3: Inverts foot through the full available range of motion.

Grade 2: Completes only partial range of motion.

For examination for Grades 1 and 0, patient is positioned sitting or supine.

Examiner sits on a low stool or stand in front of patient. Tendon of the tibialis posterior is palpated between the medial malleolus and the navicular bone. Alternatively, tendon is palpated above the malleolus.

Patient attempts to invert foot.

Grade 1: Tibialis posterior tendon will stand out if there is contractile activity in the muscle.

Grade 0: No discernible palpable contraction.

#### iv) Foot eversion with plantar flexion

The primary muscles tested are fibularis longus and brevis.

##### Grading

For examination for grades 5, 4, 3 and 2, patient is positioned sitting or supine with ankle in neutral position (midway between dorsiflexion and plantar flexion).

Examiner sits on a low stool in front of patient or stands at the end of the table if patient is supine. Patient is asked to turn foot down and out (eversion). If sufficient range is present, a stabilising hand is placed at the ankle just above the malleoli. The hand providing resistance is contoured around the dorsum and lateral border of the forefoot and directed towards inversion and slight dorsiflexion (up and in).

Patient everts foot with depression of first metatarsal head and some plantar flexion.

Grade 5: Holds test position against maximal resistance.

Grade 4: Holds test position against strong to moderate resistance.

Grade 3: Completes available range of eversion but without resistance.

Grade 2: Complete only a partial range of eversion motion.

For examination for Grades 1 and 0, patient is in a short-sitting or supine position.

Examiner sits on a low stool or stand at the end of the table. To palpate the fibularis longus, fingers are placed on the lateral leg over the upper one-third just below the head of fibula. The tendon of the muscle posterior is palpated to the lateral malleolus but behind the tendon of the fibularis brevis. To palpate the tendon of the fibularis brevis, the index finger is placed over tendon as it comes forward from behind the lateral malleolus, proximal to the base of the fifth metatarsal. The belly of the fibularis brevis can be palpated on the lateral surface of the distal leg over the fibula.

Grade 1: Contractile activity in either or both muscles, which may cause the tendon to stand out.

Grade 0: No discernible palpable contractile activity.

### Hallux and toes

- Hallux metatarsophalangeal (MP) flexion
- Toe MP flexion
- Hallux and toe distal phalanges (DIP) and proximal phalanges (PIP) flexion
- Hallux and toe MP and interphalangeal (IP) extension

#### i) Hallux MP flexion

The primary muscle tested is the flexor hallucis brevis.

##### Grading

Patient is in sitting position with legs hanging over edge of table. Alternative position is in supine position.

Examiner sits on a low stool in front of patient. Alternatively, examiner stands at side of table near patient’s foot. Test foot rests on examiner’s lap. Ask the patient to bend the big toe over finger. The index finger of the other hand is placed beneath the proximal phalanx of the great toe.

Alternatively, the tip of the finger (with very short fingernails) is placed up under the proximal phalanx.

Patient flexes great toe.

Grade 5: Holds position against strong resistance.

Grade 4: Holds test position against moderate to mild resistance.

Grade 3: Completes available range of MP flexion of great toe without resistance.

Grade 2: Completes only partial range of motion.

Grade 1: Contractile activity noted but no toe motion.

Grade 0: No discernible contractile activity.

#### ii) Toe MP flexion

The primary muscles tested are lumbricales and interossei.

##### Grading

Patient in sitting position with foot on examiner’s lap. Alternative position is supine. Ankle is in neutral (midway between dorsiflexion and plantar flexion).

Examiner sits on a low stool in front of patient. Alternatively, examiner stands next to table beside test foot. The patient is asked to bend the toes over examiner’s fingers. If sufficient range is present, a stabilising hand is placed over the dorsum of the foot (as in test for flexion of the hallux). The index finger of the other hand is placed under the MP joints of the four lateral toes to provide resistance to flexion.

Patient flexes lateral four toes at MP joints, keeping IP joints neutral.

Grade 5: Holds position against strong resistance.

Grade 4: Holds test position against moderate to mild resistance.

Grade 3: Completes available range of MP flexion of great toe without resistance.

Grade 2: Completes only partial range of motion.

Grade 1: Contractile activity noted but no toe motion.

Grade 0: No discernible contractile activity.

#### iii) Hallux and toe DIP and PIP flexion

The primary muscles tested are flexor digitorum longus, flexor digitorum brevis and flexor hallucis longus.

##### Grading

Patient is positioned sitting with foot on examiner’s lap, or supine.

Examiner sits on a short stool in front of the patient or stands at the side of the table near the patient’s foot. Patient is asked to curl toes (or big toe). If sufficient range is present, a stabilising hand is placed over the anterior foot with the fingers placed cross the dorsum of the foot and the thumb under the PIP or DIP or under the IP of hallux. Resistance is applied using four fingers or thumb of the other hand under the middle phalanges (for the IP test), under distal phalanges for the DIP test and with index finger under the distal phalanx of hallux. Resistance will be minimal.

Patient flexes the toes or hallux.

Grade 5 and Grade 4: Holds test position of toes and then hallux; resistance in both tests may be minimal.

Grade 3: Holds test position without resistance.

Grade 2: Completes only a partial range of motion.

Grade 1 and Grade 0: Minimal to no palpable contractile activity occurs. Tendon of the flexor hallucis longus may be palpated on the plantar surface of the proximal phalanx of the great toe.

#### iv) Hallux and toe MP and IP extension

The primary muscles tested are extensor digitorum longus and brevis and extensor hallucis longus.

##### Grading

Patient is positioned sitting with foot on examiner’s lap or alternatively in supine position. Ankle in neutral (midway between plantar flexion and dorsiflexion).

Examiner sits on a low stool in front of patient, or stands beside table near the patient’s foot. Patient is asked to straighten big toe or all the toes.

Patient extends lateral four toes or extends hallux.

Lateral toes: One hand stabilises the metatarsals with the fingers on the plantar surface and the thumb on the dorsum of the foot. The other hand is used to give resistance with the thumb placed over the dorsal surface of the proximal phalanges of the toes.

Hallux: The metatarsal area is stabilised by contouring the hand around the plantar surface of the foot with the thumb curving around to the base of the hallux. The other hand stabilises the foot at the heel. Place thumb over MP or IP joint for resistance.

Grade 5 and Grade 4: Toes extended fully against variable resistance (which may be small).

Grade 3: Completes range of motion with no resistance.

Grade 2: Completes a partial range of motion.

For examination for Grades 1 and 0, tendons of extensor digitorum longus are palpated or are observed over dorsum of metatarsals. Tendon of extensor digitorum brevis can be palpated on the lateral side of the dorsum of the foot just in front of the malleolus.

Grade 1: Palpable contractile activity.

Grade 0: No discernible palpable contractile activity.

## Discussion

An assessment of muscle strength is typically performed as part of a patient’s objective assessment and is an important component of the physical exam that can reveal information about neurologic deficits. It is used to evaluate weakness and can be effective in differentiating true weakness from imbalance or poor endurance. It may be referred to as motor testing, muscle strength grading or manual muscle testing. Performance of accurate muscle testing has always presented a challenge for medical students and clinicians. By referring to the examination methods mentioned in our text and with the help of related video, it is our aim to improve the quality of neurological examination among medical personnel so that diseases are recognised and managed earlier in their course.

Manual muscle testing is the most commonly used method for documenting impairments in muscle strength (1, 2). This manual test evaluates the ability of the nervous system to adapt a muscle to meet the changing pressure of the examiner’s test. The examiner should be well versed with the anatomy, physiology and neurology of muscle function. The action of the muscle being tested, as well as the role of synergistic muscles, must be fully comprehended. Manual muscle testing is both a science and an art. To produce accurate results, muscle tests must be performed according to a precise testing protocol. There are various factors that must be carefully considered when testing muscles in clinical and research settings: proper positioning in order that the test muscle is the prime mover; adequate stabilisation of regional anatomy; observation of the manner in which the patient or subject assumes and maintains the test position; observation of the manner in which the patient or subject performs the test; consistency of timing, pressure position; nonpainful execution of the test, and finally, consideration of contraindications due to age, debilitative disease, acute pain and local pathology or inflammation (2).

Muscle function can be objectified by biomechanical measurements. The most commonly held viewpoint is that the MMT (Manual Muscle Testing) is an attempt to assess the maximum force a muscle is capable of generating (3, 4). One of the most common methods used in rehabilitation to measure maximal muscle strength is isokinetic dynamometry, which provides high objectivity and reproducibility. However, the MMT that we conducted did not involve any use of specific or special tools. In fact, this method can be performed very quickly and provides flexible testing for a great number of muscles within a short period of time.

## Conclusion

The neurologic examination requires skill, intelligence and patience. It requires accurate and trained observation, performed with the help and cooperation of the patient. The examination should be carried out in an orderly manner, and adequate time and attention are necessary if the details are to be appreciated (5). Performance of accurate muscle testing has always presented a challenge for medical students and clinicians. Through reference to the lower limb examination method that we conducted and with the help of related video, our aim is to improve the quality of neurological examination among medical personnel so that diseases are recognised and managed earlier in their course.
